# Meiotic failure in cyclin A1-deficient mouse spermatocytes triggers apoptosis through intrinsic and extrinsic signaling pathways and 14-3-3 proteins

**DOI:** 10.1371/journal.pone.0173926

**Published:** 2017-03-16

**Authors:** Sunil K. Panigrahi, Marcia Manterola, Debra J. Wolgemuth

**Affiliations:** 1 Departments of Genetics & Development, Columbia University Medical Center, New York, New York, United States of America; 2 Program of Human Genetics, ICBM, Faculty of Medicine, University of Chile, Santiago, Chile; 3 Obstetrics & Gynecology, Columbia University Medical Center, New York, New York, United States of America; 4 Institute of Human Nutrition, Columbia University Medical Center, New York, New York, United States of America; 5 Herbert Irving Comprehensive Cancer Center, Columbia University Medical Center, New York, New York, United States of America; University Hospital of Münster, GERMANY

## Abstract

Cyclin A1 (Ccna1), a member of the mammalian A type cyclins, is most abundantly expressed in spermatocytes and is essential for spermatogenesis in the mouse. Ccna1- deficient spermatocytes arrest at late meiotic prophase and undergo apoptosis. To further delineate the mechanisms and key factors involved in this process, we have examined changes in expression of genes involved in both intrinsic and extrinsic signaling pathways that trigger apoptosis in the mutant spermatocytes. Our results show that both pathways are involved, and that the factors involved in the intrinsic pathway were expressed earlier than those involved in the extrinsic pathway. We have also begun to identify *in vivo* Ccna1-interacting proteins, using an unbiased biochemical approach, and identified 14-3-3, a key regulator of apoptosis, as a Ccna1-interacting protein. Expression levels of 14-3-3 proteins remain unchanged between wild type and mutant testes but there were differences in the subcellular distribution. In wild type control, 14-3-3 is detected in both cytosolic and nuclear fractions whereas it is restricted to the cytoplasm in mutant testes. This differential distribution of 14-3-3 may contribute to the induction of apoptosis in Ccna1-deficient spermatocytes. These results provide insight into the apoptotic mechanisms and pathways that are triggered when progression through the meiotic cell cycle is defective in male gametogenesis.

## Introduction

Apoptosis is an evolutionarily conserved mechanism of programed cell death that occurs in response to certain physiological stimuli, cell injury, or stress, and is a vital component of various developmental processes in metazoans [1, 2]. In the mammalian testis, apoptosis plays a major role in regulating spermatogenesis [3] and in ensuring the genetic quality of the gamete [4]. In juvenile mouse testes, massive apoptosis occurs during the first wave of spermatogenesis to regulate the number of germ cells and their ratio relative to the number of Sertoli cells [5]. During meiosis, apoptosis is triggered when defects in complex processes such as synapsis, recombination, and segregation of homologues compromise the quality of the spermatocyte [6]. Indeed, the normal occurrence of these processes is strictly regulated by meiotic checkpoints that create a network of signaling mechanisms that modulates the activity and progression of meiosis. Depletion of essential survival factors and persistent meiotic defects are sensed by these highly conserved meiotic checkpoints, which then cause an arrest of cellular progression of the defective cells and an induction of the apoptotic program [7].

The regulation of apoptosis in the various spermatogenic cells involves both an extrinsic apoptotic pathway (cell surface death receptor-mediated) involving the Fas/FasL proteins and caspases 8 and/or 10 [3] as well as an intrinsic pathway (mitochondria-mediated) through the Bax/Bcl-2 family proteins and caspase 9 [8]. In meiosis, apoptosis is generally triggered by a CHK2-dependent activation of the p53 family proteins in specific sub-stages of prophase I [9]. There is an interesting sexual dichotomy in terms of how stringently apoptosis is induced during meiosis. That is, during oogenesis, not all meiotic defects drive cells to undergo apoptosis, making oocytes more susceptible to completing meiosis carrying chromosome defects [10]. When apoptosis does occur, both intrinsic and extrinsic pathways are involved in the death of the oocytes at various stages of meiotic prophase [11]. In contrast, spermatocytes with meiotic defects usually undergo apoptosis during pachynema, the transition of prophase I to metaphase I, and/or metaphase I [12]. Despite significant progress in the understanding of the meiotic checkpoints, little is known about the apoptotic mechanisms and pathways that are triggered when meiosis is defective in male gametogenesis.

We have shown by targeted mutagenesis that the male germ cell specific A-type cyclin, cyclin A1 (Ccna1), is an essential regulator of the transition of spermatocytes from prophase I to metaphase I [13]. Deficiency of Ccna1 resulted in a reduction of Cdk2 activation at the end of meiotic prophase I, failure to progress into metaphase I, and induction of apoptosis in late prophase spermatocytes [14, 15]. We have shown that this apoptosis is mediated by p53-dependent and notably, also p53-independent pathways [15]. We also observed an activation of caspase-3 and an increase in the levels of and change of subcellular localization of the apoptotic factor Bax [15].

In the present work, we extend these studies by showing that the apoptosis induced by the depletion of Ccna1 in male meiosis involves changes in the ratio of apoptotic agonists and antagonists in the defective spermatocytes. That is, loss of Ccna1 initiates apoptosis in spermatocytes both by up-regulating pro-apoptotic genes and down-regulating anti-apoptotic genes. We show that these changes in gene expression appear to be meiotic-stage specific, as that the intrinsic apoptotic pathway is triggered in mutant spermatocytes before the activation of the extrinsic pathway. Notably, we also identify 14-3-3 proteins as novel Ccna1 interacting partners and show that their subcellular distribution is altered in the absence of Ccna1. Our results suggest that interaction of Ccna1 with 14-3-3 is important for meiotic progression as well as inhibition of apoptosis in spermatocytes.

## Materials and methods

### Experimental animals

The Institutional Animal Care and Use Committee (IACUC) of Columbia University Medical Center, New York, NY, USA approved all procedures involving animals. All animals were housed in a pathogen free barrier facility and all manipulations were performed in accordance with IACUC guidelines. *Ccna1*^+/-^ males were mated with *Ccna1*^-/-^ females to generate *Ccna1*^-/-^ males and in parallel, *Ccna1*^+/+^ males and females of same genetic background were mated to obtain *Ccna1*^+/+^ (referred as WT throughout) males for this study. Testes from *Ccna1*^-/-^ and the corresponding WT animals of postnatal day (PND) 17, 22 and 28 were used in the gene expression and immunolocalization analyses. For pull-down and immunoprecipitation (IP) experiments, WT mice of 8 weeks of age were used.

### Sub-cellular fractionation and germ cell separation

The preparation of single cell suspensions from testes was performed according to our laboratory’s established protocol [16]. Total cell lysates from testicular suspensions were made as previously described [17]. Nuclear and cytoplasmic fractions from testicular cells were prepared using the Active Motif nuclear extract kit (Active Motif Inc., #40010) according to the manufacturer’s protocol. Mitochondrial protein lysate was prepared by using the Thermo Scientific mitochondria isolation kit (Thermo Scientific, #89874). Preparation of enriched populations of pachytene spermatocytes was carried out according to our laboratory’s established protocol [16]. The purity of cell populations was confirmed by flow-cytometry as described [18]. Protein concentrations in total testicular lysates, pachytene cell lysates and subcellular lysates were determined by the Bradford assay (Bio-Rad., # 500–0006).

### RNA purification and real-time PCR array

Preparation of testicular tubule suspensions from testes was carried out according to our laboratory’s established protocol [16]. Total RNA was isolated from eighteen individual mouse testes (three pairs of testes for age group and genotype as noted above) using RT² qPCR-Grade RNA Isolation Kit (Super Array Bioscience Corp., # PA001) as per the manufacturer’s protocol. Total RNA was rendered free of genomic DNA by on-column digestion with RNase-free DNase1. Gene expression analysis for apoptotic pathways was performed using a real time-PCR array (qRT-PCR) for mouse apoptosis (SuperArray Bioscience Corp., # PAMM012Z) containing 84 apoptotis-related genes as described for mouse apoptosis in the RT^2^ Profiler PCR Arrays list (http://www.sabiosciences.com/rt_pcr_product/HTML/PAMM-012Z.html, Qiagen). qRT-PCR analysis of gene expression was carried out in an ABI 7300 gene expression system (Applied Bio Systems). Real-time data were analyzed using the support system provided by the manufacturer and values plotted in Geom plot using R software. In brief, the mean Ct value of three independent experiments was uploaded to the browser for each set of experiments. Analysis was undertaken using the basic “set up” criteria and the quality was reviewed by “Data QC” to ascertain the PCR reproducibility, reverse transcription efficiency, and genomic DNA contamination. After passing the Data QC, each data set was normalized to housekeeping genes already in the “set up”. The analyzed data provided the fold change or fold regulation of the mutant group as compared to the control group for each set of experiments. In our case it was for each age group; PND 17, PND 22 and PND 28 respectively.

Five genes identified in the array analysis were selected for confirmation and validation by qRT-PCR. We picked at least two genes from each apoptotic pathway; either intrinsic or extrinsic for further confirmation. In brief, 500ng of total RNA from each sample was reverse transcribed using Superscript III first strand synthesis system (Invitrogen Inc. #18080–051) with oligo dT primer in a 20μl reaction volume. Equal amounts of cDNA were subjected to real-time PCR using SYBR-Green PCR master mix (Applied Bio systems) in an ABI 7300 qRT-PCR system. Primer pairs used in this study were as follows: *Bcl2l10*, 5’- CCACTGCATGAACGCACTAGA-3’ (forward) and 5’-GAGCAACTTATCTGCCATCTCG-3’ (reverse); *Cradd*, 5’-AAGGCGAGAGAGGA AGTCACA (forward) and 5’-GTTAATCTGCTGGTCTGATGGC-3’ (reverse); *Fas*, 5’-TATCAAGGAGGCCCATTTTGC-3’ (forward) and 5’-TGTTTCCACTTCTAAA CCATGCT-3’ (reverse); *p21*, 5’-CCTGGTGATGTCCGACCTG-3’ (forward) and 5’-CCATGAGCGCATCGCAATC-3’ (reverse); *Sphk2*, 5’-CACGGCGAGTTTGGTTCCT A-3’ (forward) and 5’- CTTCTGGCTTTGGGCGTCGT-3’ (reverse); *Tnf*, 5’-TTGGTGG TTTGTGAGTGTGAG-3’ (forward) and 5’- GACGTGGAACTGGCAGAAGAG-3’ (reverse); and *Arbp*, 5’- CAAAGCTGAAGCAAAGGAAGAG-3’ (forward) and 5’-AATTAAGCAGGCTGACTTGGTTG-3’ (reverse).

### FLAG-Ccna1 pull down

FLAG peptide was fused to full-length *Ccna1* cDNA sequence, inserted into the pFastBac1 donor plasmid (Invitrogen #10359–016), and then transformed to DH10Bac *E*. *coli* cells to produce recombinant bacmid. Recombinant bacmid containing FLAG-Ccna1 was transfected into sf9 cells using CELLFECTIN reagent (Invitrogen Inc., #10362). Recombinant baculovirus particles were isolated and infected into fresh sf9 cells for protein expression. FLAG-Ccna1 was purified from 72 hrs post-infected cells using Anti-FLAG M2 agarose beads (Sigma Aldrich, #2220) according to the manufacturer’s instructions. For the pull down experiment, 1mg of lysate from enriched pachytene cells was incubated with immobilized FLAG-Ccna1 overnight at 4°C with rotation. A control experiment was performed in parallel, where equal amount of lysates were incubated with immobilized FLAG peptide only. The immobilized beads were washed three times with 1×TBST (0.9% NaCl, 0.1% Tween- 20, 100 mM Tris-HCl, pH 7.5) and once with 1×TBS. Bound proteins were extracted by boiling the beads with 1×SDS loading buffer for 5 minutes. Eluted proteins were subjected to SDS-PAGE for 15 minutes. Once they entered into the resolving gel, the current was turned off. Gel bands (1cm^2^) were cut and sent to the Columbia University protein core facility for mass spectrometry analysis (LC-MS/MS). Raw data from mass spectrometry were acquired by Mass Lynx and then converted to text files by using Protein Lynx software. A database search was performed using reference sequence databases from *Mus musculus*. Proteins/peptides were identified by using the mascot algorithm at www.matrixscience.com.

### Preparation of testes lysate, immunoprecipitation and immunoblotting

For immunoprecipitation and analysis, total lysates from whole testes of WT adult animals were used. For, immunoblot analysis lysates were obtained from both WT and mutant testes. Total testes lysates were prepared as described [17]. In brief, dissected and decapsulated testes were homogenized immediately in RIPA buffer (50 mM Tris-Cl pH7.5, 150 mM NaCl, 0.1% SDS, 0.5% Na deoxycholate, 1% NP-40 and protease inhibitor cocktail from Sigma) with a tissue tearor (BioSpec Products Inc, Model #985370). Insoluble material was removed by centrifugation for 20 minutes at 12,000×g, at 4°C. Protein concentration of the supernatants was measured using the Bradford assay (Bio-Rad., # 500–0006). A total of 1mg of protein was used for IP with antibodies against 14-3-3, Ccna1, or Rabbit IgG at 4°C overnight. For direct immunoblot, the lysates (25μg/lane) were subjected to electrophoresis on SDS-polyacrylamide gels. Proteins were transferred to polyvinylidene fluoride membranes blocked with 5% nonfat milk in 1× TBST and incubated with primary antibodies overnight at 4°C. Primary antibodies used in this study are as follows: p21, Santa Cruz Biotechnology # sc-397; 14-3-3, Epitomics # 5175–1; Caspase 8, Cell Signaling # 4927; Caspase 9, Cell Signaling # 9504; Caspase 10, Cell Signaling # 9752; Bax, Santa Cruz Biotechnology # sc493; Bad, Santa Cruz Biotechnology # sc943; Phospho-Akt, Cell Signaling # 4060; Akt, Cell Signaling # 2920; and Ccna1 (generated by our lab). After three washes with TBST, the membranes were incubated with horseradish peroxidase (HRP) conjugated rabbit/mouse secondary antibodies (Santa Cruz Biotechnology # sc2004 and # sc2005) for 1 hour. After three additional washes with 1× TBST, HRP activity was visualized with the Millipore Immobilon Western Blotting Detection kit (Millipore, #WBKLS0500) according to the manufacturer’s instructions. Band intensity was quantified using image J software and normalized with loading controls.

### Immunofluorescence

Testes from 17 day-old WT and *Ccna1*^-/-^ mice were embedded in OCT, after fixation with 4% paraformaldehyde (PFA) and cryopreservation in 30% sucrose. Frozen sections were cut and mounted on glass slides. For immunostaining, the following primary antibodies were used: mouse anti-caspase 10, clone 16C5.1 (1:100; EMD Millipore, # MABC192); mouse anti-cleaved caspase 9 (1:100; Cell Signaling, # 9509S); and guinea pig anti-histone H1t (1:500; kindly provided by Mary Ann Handel). After rinsing in PBST, the slides were incubated with appropriate secondary antibodies diluted 1:200 in 1× PBS; Alexa 488-conjugated donkey anti-mouse IgG (H+L) and Alexa 594-conjugated donkey anti-guinea pig IgG (H+L) (Jackson Immuno Research, # 706-586-148). Slides were counter-stained with DAPI and mounted with Prolong Gold (Thermo Fisher Scientific, # P36930). Slides were examined using a Nikon eclipse E800 microscope equipped with epifluorescence optics and the images were photographed with a high-definition cooled color camera head DS-Fi1c. All images were processed with Adobe Photoshop CS5 software.

To determine the number and percentage of late prophase I spermatocytes that were caspase 9- or caspase 10-positive in WT and *Ccna1*^-/-^ testes, testicular sections from three different mice per genotype were examined and an average of 400 cells positive for H1t per animal, which correspond to mid-pachytene to diplotene spermatocytes, were counted. From the H1t positive cells, we then quantified the number of cells that were positive for caspase 9 or caspase 10. The quantification is presented as percentage ± standard deviation (SD). A statistical t test was performed using a nonparametric Mann-Whitney signed-rank test to determine the difference between the genotypes, and the threshold of significance was set at 0.05 (GraphPad Prism 5, GraphPad Software, Inc.).

### Immunohistochemistry

Testes of mice at 17, 22 and 28 days of age were dissected and fixed with 4% paraformaldehyde overnight at 4°C. Fixed tissues were embedded in paraffin, sectioned at 5μm-thick and mounted on slides. Immunohistochemistry was performed using a Vectastain ABC kit for Rabbit IgG (# PK-6101, Vector Laboratories, Burlingame, CA) as described [13]. In brief, histological sections were deparaffinized in histoclear, hydrated through an alcohol series followed by a washing with distilled water. Antigen retrival was performed by boiling the slides in a microwave for 10 min in 0.01M-citrate buffer, pH 6.0. Endogenous peroxidase was inactivated by incubating the slides with 3% hydrogen peroxide for 30 minutes and then incubated with blocking solution (2.5% goat serum) for 1 hr at room temperature. The sections were then incubated with rabbit- anti Phospho-Akt Ser 473 (Cell Signaling # 4060) at 1:50 dilution overnight at 4°C. After three washes in 1XTBST (TBS with 0.1% TritonX-100), subsequent steps were performed according to the manufacturer’s instructions. For visualization, the slides were incubated with diaminobenzidine solution for 5 min, washed 3 times with distilled water and counter stained with hematoxylin. Sections were dehydrated through an alcohol series and mounted with permount mounting medium (Fisher Scientific # SP15-100). Slides were examined on a Nikon microscope under brightfield optics and the images were processed using Adobe Photoshop CS5 (Adobe, San Jose, CA).

## Results and discussion

### Differential expression of apoptosis related genes in *Ccna1*^-/-^ spermatocytes

To identify the factors and pathways involved in the induction of apoptosis that occurs in late prophase *Ccna1*^-/-^ spermatocytes [13], we performed a qRT-PCR array analysis using the Super Array (SA biosciences, # PAMM012Z), which measures the expression of 84 apoptosis-related mouse genes. RNA samples were obtained from WT and *Ccna1*^*-/-*^ testicular tubule suspensions at PND 17, 22, and 28. These three time points were chosen based on previous TUNEL assays showing that in the mutant testes, there were few apoptotic spermatocytes at PND 17, that they increased significantly at PND 22, and that they were robustly detected at PND 28 [15]. At PND 17, the cells in the first wave of spermatogenesis and would have reached the pachytene-diplotene stage of meiosis. So theoretically, at this age both WT and mutant testes have a similar cellular composition. At PND 22 and PND 28, WT testes reached the spermatid stages and contain spermatogenic cells from spermatogonia to spermatids. In *Ccna1*^-/-^ mutant testes, spermatocytes are arrested at mid-diplotene stage and hence do not contain spermatids. Although the cellular composition is different at PND 22 and PND 28 between the WT and *Ccna1*^-/-^ testes, the spermatids in the WT tubules are not TUNEL positive cells [15]. We therefore suggest that the presence of spermatids in the WT testes will not affect the expression of apoptosis-related genes and detection of changes in expression in our array analysis.

At PND 17, we found only two genes in the array, which were modulated in *Ccna1*^-/-^ tubules as compared to the WT control. *Nod1* and *Card6* were both down regulated by 3.03- and 5.88-fold, respectively, and no genes were up regulated at this age (S1A Fig; S1 Table). At PND 22, 39% (33 out of 84 genes) of the analyzed targets exhibited a change in expression (S1B Fig; S1 Table): 19 were up regulated and 14 were down regulated. At PND 28, the number of differentially expressed genes between WT and mutant was reduced to 28% (23 of 84 genes): 21 genes were up regulated and only two were down regulated (S1C Fig; S1 Table).

This trend of expression of pro-apoptotic genes was tightly correlated to the expression pattern and function of Ccna1 protein during meiosis. That is, at PND 17, most of the spermatocytes are at late pachytene and diplotene stages and thus, are robustly expressing Ccna1. In the absence of Ccna1, the defects produced by its depletion will have just started to manifest and the induction to undergo apoptosis will have been initiated in only a few cells. However, by PND 22, most of the late pachytene and diplotene spermatocytes will have initiated apoptosis and exhibit TUNEL positive staining with all apoptotic pathways activated [15]. This observation is also consistent with our previous findings of no significant increase in TUNEL positive cells in the *Ccna1*^-/-^ testes at PND 17 but massive TUNEL-positive cells at PND 22 [15].

Although we previously found that the number of TUNEL-positive cells remains elevated in the PND 28 mutant testes [15], the number of apoptosis-related gene expression changes was reduced to 23, compared to the 33 genes observed in PND 22 (S1 Table). This difference is likely due to the fact that the signaling to enter apoptosis was no longer needed and the TUNEL-positive staining was due to non-clearance of apoptotic cells at this age. Interestingly, at PND 28 there were many more up-regulated genes (21) relative to down-regulated genes (2). Indeed, the majority of the up-regulated genes seen in PND 22 were still up-regulated at PND 28, while most of the down-regulated genes seen at PND 22 were no longer repressed at PND 28 (S1 Table). These results suggest that induction of apoptosis as a consequence of the absence of Ccna1 is initiated by both up regulating pro-apoptotic genes, such as *Bcl2l10*, *Card6*, *Card10*, *Casp4*, *Fas* and *Tnf-α*, and down-regulating anti-apoptotic genes, such as *Bcl2l1*, *Birc2*, *Trp63* and notably, *Trp53*. This observation reinforced the idea that although late pachytene and diplotene spermatocytes may have sensed the absence of Ccna1, apoptotic signals at PND 17 are not massively triggered. These observations further suggest that at later stages of the apoptotic response, the up-regulation of pro-apoptotic genes might be more important than decreased expression of anti-apoptotic genes.

**Fig 1:**
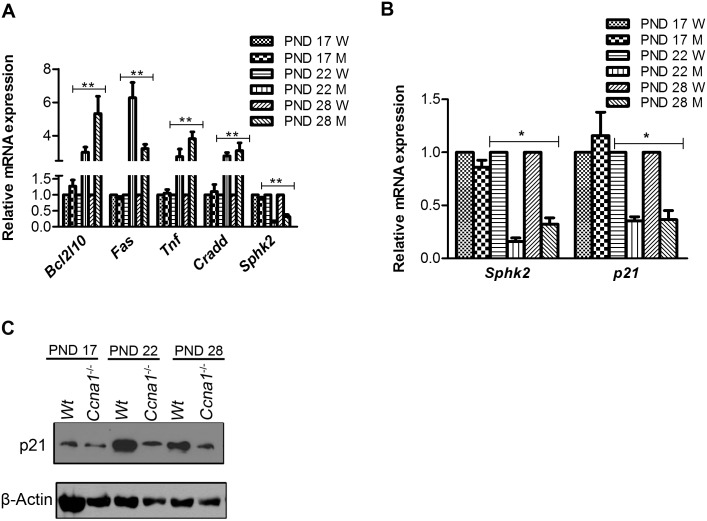
Sphk2 mediated down-regulation of p21 in *Ccna1*^*-/-*^ mutant testes. A. Validation of relative mRNA expression of selected genes from the array analysis, namely *Bcl2l1*, *Fas*, *Tnf*, *Cradd* and *Sphk2*, in WT (W) and *Ccna1*^*-/-*^ (M) testicular tubule suspensions as determined by qRT-PCR. B and C. The expression of *Sphk2* and its possible target *p21* was measured at both RNA (panel B) and protein (panel C) levels at indicated stages of testes development i.e. PND 17, PND 22 and PND 28 respectively. *Arbp* was used as internal control for qRT-PCR and β-Actin served as loading control in the immunoblot. Error bars represent standard deviation. * p< 0.05 and ** p< 0.01.

To confirm and validate the data obtained with the array analysis, five genes were chosen randomly (three for the intrinsic pathway and two for the extrinsic pathway specific) and their mRNA levels examined by qRT-PCR using different sets of primers from the array analysis and normalized to the housekeeping gene *Arbp* (which is different from actin and GAPDH that were used in the Super Array) in WT and *Ccna1*^-/-^ testes at PND 17, PND 22 and PND 28 (Fig 1A). A similar pattern of change in gene expression was observed as compared to the array analysis.

In the array analysis, we noted that the expression of an anti-apoptotic gene, sphingosine kinase 2 (*Sphk2*), was down regulated in *Ccna1*^-/-^ testes at both PND 22 and 28, but not at PND 17 (S1 Table). This is of interest, as *Sphk2* is up regulated in several cancers and down regulation of *Sphk2* activates apoptotic proteins by several pathways including EGFR and PI3K/AKT signaling [19, 20]. Moreover targeted siRNA knock down of *Sphk2* has been reported to result in the down-regulation of p21 in a p53-independent manner [21]. Interestingly, our previous study showed that deletion of p53 could not rescue the apoptosis in *Ccna1*^-/-^ testes [15]. It is thus possible that the down-regulation of Sphk2 in the absence of Ccna1 in our model induces a down-regulation of p21 and subsequent progression of apoptosis. Indeed, our qRT-PCR analysis revealed that both *Sphk2* and *p21* mRNA levels were reduced in the mutant testes at PND 22 and PND 28, but not significantly at PND 17, relative to WT control (Fig 1B). Immunoblot analysis further revealed a decrease in p21 protein levels in *Ccna1*^-/-^ testes compared to WT (Fig 1C). This suggests that Sphk2 could act as a regulator of p21 expression and that loss of Ccna1 function results in a decrease in the expression of Sphk2 and subsequently, p21. Such deregulation could contribute to impede the pro-survival activity in *Ccna1*^-/-^ spermatocytes, leading to the progression of apoptosis.

### Intrinsic apoptotic signaling is activated earlier than extrinsic signaling in Ccna1-deficient spermatocytes

Our results from the array analysis showed modulation of both pro- and anti-apoptotic genes in Ccna1-deficient testes and importantly, we noted that these factors were from both intrinsic (*Bcl2l1*, *Bcl2l10*, *Bric2*, *Trp53* and *Sphk2*) and extrinsic (*Tnf*, *Fas* and *Il10*) pathways (S1 Table). Bcl family members are known to initiate the intrinsic pathway by activating the initiator caspase 9, while death domain receptors like Fas and Tnf execute the extrinsic apoptosis pathway by recruiting caspases 8 and/or 10 as initiator molecules. In the array analysis, caspase 9 was expressed at 1.95- and 1.68-fold higher levels in mutant testes as compared to the WT at PND 22 and PND 28, respectively (S1 Table). As we defined a cut-off of ±3 fold in our array data analysis, caspase 9 was not highlighted in the table and no change in caspase 8 expression was observed. Unfortunately, caspase 10 was not included in the Super Array.

**Fig 2:**
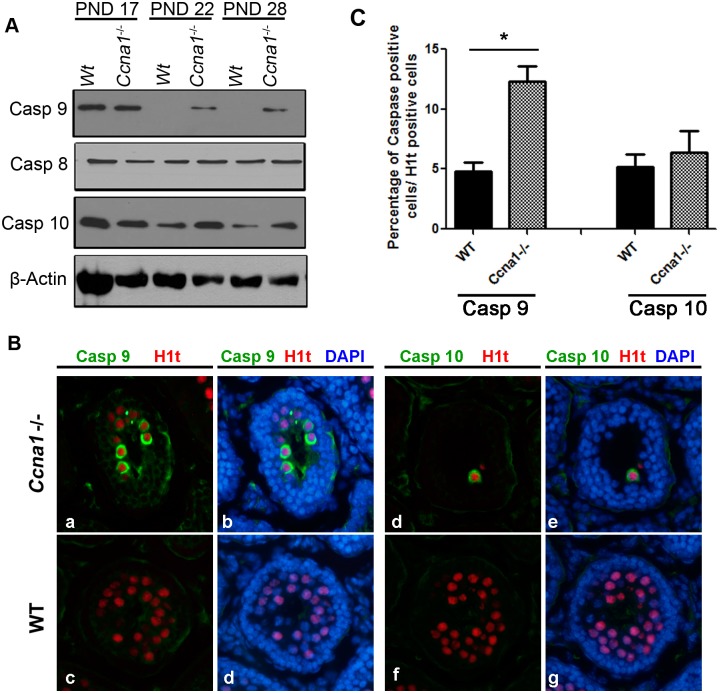
Activation of the intrinsic apoptosis pathway occurs earlier than that of the extrinsic pathway in Ccna1-deficient testes. A. Markers of intrinsic (caspase 9) and extrinsic (caspase 8 and 10) pathways were analyzed by immunoblot analysis using testicular lysates from WT and Ccna1-deficient mice at PND 17, 22 and 28. β-Actin served as loading control. B. Immunostaining of caspase 9 and 10 in both WT and mutant histological sections of PND 17 testes. H1t was used as a marker for accurate classification of late prophase I spermatocytes (c-d, f-g). C. Quantification of late prophase I spermatocytes that were positive for caspase 9 and 10 in WT and mutant testes at PND 17. Error bars represent standard deviation. * p< 0.05.

To confirm that both intrinsic and extrinsic apoptotic pathways are involved in the apoptosis of *Ccna1*^-/-^ spermatocytes, we examined the protein levels of the intrinsic pathway initiator caspase 9 and the extrinsic pathway initiators caspase 8 and 10. Caspase 9 was undetectable and caspase 10 levels were low in WT testis at PND 22 and PND 28, which correlated with our previous observations of absence of TUNEL positive cells in WT cells at these time points [15]. Caspase 8 protein levels were unchanged in the testis lysates irrespective of the age and genotype of the animals (Fig 2A), which was consistent with the array data. In contrast, while no significant changes were detected at PND 17, we observed an increase in the levels of both caspase 9 and 10 in *Ccna1*^-/-^ lysates at PND 22 and PND 28 (Fig 2A).

We then asked which apoptotic pathway is triggered first in the absence of Ccna1 in spermatocytes. To define the presence of intrinsic or extrinsic pathways at the single spermatocyte level, we use immunofluorescence detection of the initiator caspases, caspase 9 (cleaved form) and caspase 10 (total), along with detection of histone H1t to precisely identify late prophase I spermatocytes. We first examined testes from PND 22 mice; however, both caspases were co-expressed in most tubules and thus, identifying the temporal appearance of each pathway was not possible at this age (data not shown). We therefore examined testes from PND 17 mice. Mice of this age allowed us to detect the first diplotene cells that are undergoing apoptosis in response to Ccna1 depletion. We found that the intrinsic pathway initiator caspase, caspase 9, appeared in mid/late pachytene spermatocytes (as identified by being H1t-positive) at PND 17 and at this stage, there was little if any staining for caspase 10 (Fig 2B). Quantification of the stained cells revealed that 12% ± 4.3 of late prophase I cells in *Ccna1*^-/-^ testes were positive for caspase 9 as compared to 4% ± 2.1 observed in the WT control. No significant differences in caspase 10 signals were observed between PND 17 WT and *Ccna1*^*-/-*^ testes (Fig 2C). These results indicate that activation of the intrinsic apoptotic pathway is an earlier event in response to the absence of Ccna1 in spermatocytes, and precedes the extrinsic pathway function.

### Identification of 14-3-3 proteins as Ccna1 interacting proteins

We next considered that there might be apoptotic-signaling molecules that actually interact with and are inhibited by Ccna1 and which would be activated in its absence. To identify Ccna1-interacting proteins in testis, we used a FLAG-pull down approach by expressing and purifying FLAG-tagged Ccna1 from sf9 cells. Ccna1-interacting proteins were then purified from lysates of purified WT pachytene spermatocytes using immobilized FLAG-Ccna1 beads and FLAG-M2 beads as control. Bound proteins were eluted, and identified by LC-MS-MS as described in materials and methods. With this approach, we identified a total of 30 candidate proteins that bound specifically to Ccna1 but not to control beads (S2 Table).

**Fig 3:**
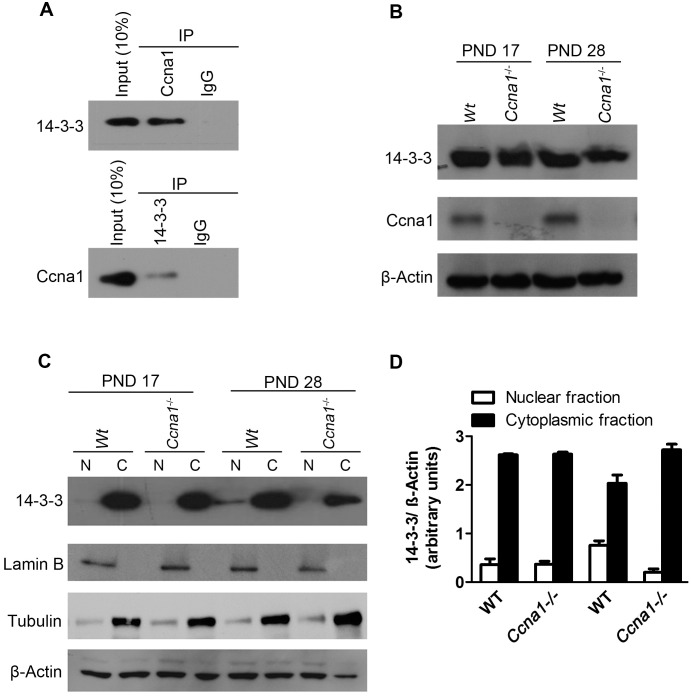
Ccna1 interacts with the apoptotic inhibitor 14-3-3 and modulates its subcellular localization. A. Reciprocal immunoprecipitation using either anti-14-3-3 (upper panel) or Ccna1 (lower panel) antibodies confirmed the interaction of Ccna1 with 14-3-3 in adult mouse testes. B. The overall levels of 14-3-3 proteins did not change in mutant versus WT testes as analyzed by immunoblot analysis in PND 17 and PND 28 testicular lysates. β-Actin served as loading control. C. The sub-cellular distribution of 14-3-3 proteins was altered in Ccna1-deficient as compared to WT testes at PND 17 and 28 as analyzed by immunoblot analysis using lysates from cytoplasmic (C) and nuclear fractions (N). Lamin B and tubulin were used as markers for purity of nuclear and cytoplasmic fractions, respectively. β -Actin was used as the loading control. D. Quantification of 14-3-3 levels in both cytoplasmic and nuclear fractions of PND 17 and PND 28 from two independent experiments. Error bars represent standard deviation.

The presence of the 14-3-3 proteins in this list was of particular interest as 14-3-3 proteins are involved in various physiological processes including the inhibition of apoptosis [22, 23]. Mammalian 14-3-3- proteins consist of a family of 7 isoforms [24]. Our mass spectrometry analysis identified two isoforms (ζ and θ) of 14-3-3 as binding partners of Ccna1. Three isoforms of 14-3-3 (β, θ and ε) have been reported to be expressed in testis and, interestingly, the θ and ε isoforms were expressed in testicular germ cells whereas expression of 14-3-3 β is restricted to Sertoli and Leydig cells [25]. Indeed, 14-3-3 θ is expressed in both pachytene spermatocytes and spermatids [25]. The expression pattern of ζ isoform and other isoforms in testis is unknown. Reciprocal immunoprecipitation using either anti-Ccna1 or anti-14-3-3 antibodies confirmed that Ccna1 indeed interacts with 14-3-3 proteins (Fig 3A). We next analyzed whether this interaction affects the expression levels of 14-3-3 by immunoblot analysis. Depletion of Ccna1 did not affect the protein levels of 14-3-3 in mutant testicular lysates as compared with WT (Fig 3B). While the antibody we had used recognizes all 14-3-3 isoforms, the results should reflect the θ, ζ or ε isoforms due to their expression pattern in the testis [25] and our mass spectrometry data.

### Differences in the sub-cellular distribution of the 14-3-3 proteins in the absence of Ccna1

The functions of 14-3-3 proteins vary depending on their subcellular distribution, their phosphorylation status, and the phosphorylation status of their interacting partners. In the nucleus, they have been shown to induce the translocation of transcription factors such as FOXO from the nucleus to the cytoplasm [26], thus inhibiting expression of pro-apoptotic genes like Bax, Bad, and Bim [27, 28]. The 14-3-3 proteins can also function in the cytoplasm by sequestering pro-apoptotic factors like Bax and Bad, and inhibiting their translocation to the mitochondria [29, 30]. We therefore asked whether the sub-cellular localization of 14-3-3 proteins is altered in the absence of Ccna1. Immunoblot analysis of nuclear and cytoplasmic fractions of testicular lysates (*n* = 6 mice, from 2 independent experiments) revealed a differential distribution of 14-3-3 proteins in PND28 testes but not in PND 17 testes. In WT testes, 72.4% ± 4.1 of 14-3-3 proteins were present in the cytoplasm and 27.6% ± 4.1 were found in the nuclear fraction (Fig 3C and 3D). In contrast, in *Ccna1*^-/-^ testes, 94% ± 2.3 of 14-3-3 proteins were present in the cytoplasm and only 6% ±2.3 were found in the nuclear fraction (Fig 3C and 3D). This change was not due to variations in the protein levels, as the total 14-3-3 protein levels (nuclear and cytoplasmic) remained the same between WT and mutant lysates at both PND 17 and PND 28 (Fig 3B and 3D). This result shows that in the absence of Ccna1, the subcellular localization of 14-3-3 was now mainly cytoplasmic rather than both cytoplasmic and nuclear. In addition, the decreased levels of 14-3-3 observed in the nuclear fractions of *Ccna1*^-/-^ spermatocytes suggest that the inhibition of expression of pro-apoptotic genes like Bax and Bad could be affected in the absence of Ccna1. In fact, our Super Array data showed that *Bax* and *Bad* mRNA levels were >2 fold higher in PND 28 *Ccna1*^-/-^ testes compared to WT control.

**Fig 4:**
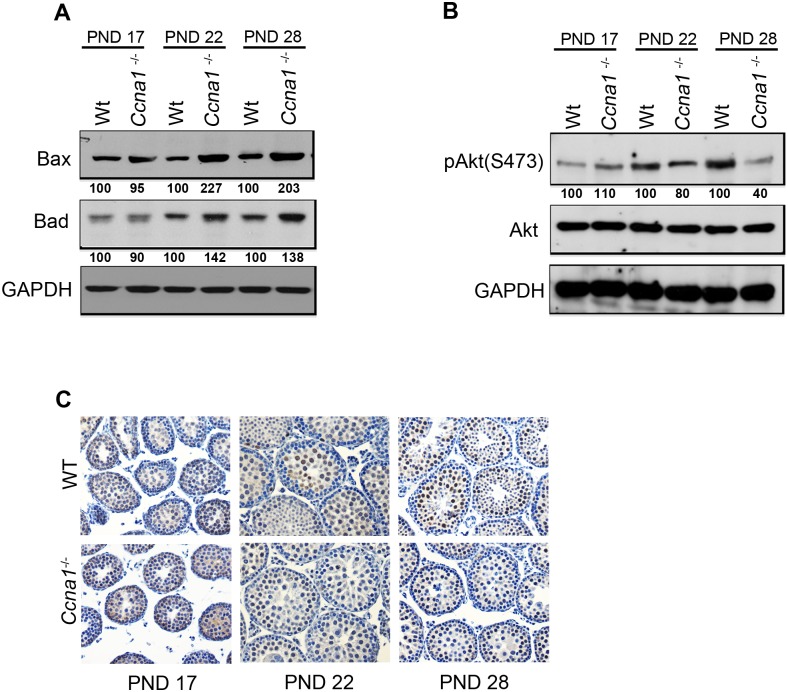
Apoptosis in Ccna1-deficient testes is characterized by elevated levels of Bax and Bad and decreased phosphorylation of Akt. A. Protein levels of Bax and Bad are increased in PND 22 and 28 Ccna1-deficient testes as compared to WT controls as analyzed by immunoblot analysis using lysates from WT and *Ccna1*^-/-^ testes at different developmental ages. GAPDH served as the loading control. B. Akt phosphorylation was decreased in the lysates *Ccna1*^-/-^ testes versus WT at different developmental ages as analyzed by immunoblot analysis. Total Akt levels were taken as the reference, and GAPDH served as the loading control. Numbers under each lane represent the relative percentage of protein levels in mutant lysate compared to the WT control (100%) at indicated postnatal days. C. Immunohistochemistry of phospho-Akt in WT and *Ccna1*^-/-^ PND 17, PND 21 and PND 28 testes. The intensity of Akt phosphorylation in *Ccna1*^-/-^ spermatocytes was decreased as compared to WT control at PND 22 and PND 28. Original magnification x40.

We next analyzed if the changes in the mRNA levels reflect the protein levels of Bax and Bad. To this end, we measured the abundance of these proteins in WT and mutant testicular lysates. We observed that the expression of both Bax and Bad was higher in mutant testes as compared to wild type control at PND 22 and 28, whereas their levels did not change in PND 17 lysates (Fig 4A). Therefore, our results suggest that depletion of Ccna1 results in an increment of Bax and Bad levels in spermatocytes and further suggest that the changes in the subcellular localization of 14-3-3 may be related to the expression of *Bax* and *Bad* in the mutant cells.

### Akt phosphorylation is impaired in Ccna1 deficient spermatocytes

It is known that 14-3-3 exerts its cell survival function by interacting with and inhibiting the pro-apoptotic function of several proteins, including Bad, FOXO, Raf1, and Death Associate Protein Kinase 2 (DAPK2) [31–34]. Phosphorylation of the above proteins is necessary for their interaction with 14-3-3 and interestingly, all of them are reported as Akt substrates. Furthermore, it has been shown that the activity of Akt fluctuates across the mitotic cell cycle and mirrors the expression of cyclin A2 [35]. Moreover, cyclin A2-Cdk complexes can phosphorylate and activate Akt [35]. It is thus possible that during normal meiosis, cyclin A1 mediates a pro-survival signaling both by regulating and activating Akt and also by regulating 14-3-3 activities.

To examine this possibility, we determined the phosphorylation status of Akt, which is known to be decreased either by depletion of the somatic A-type cyclin, cyclin A2, or CDK2 in mitotic cells [35] and also favors cell proliferation. No differences in phospho-Akt (serine 473) levels were observed at PND 17 (Fig 4B). However, Akt phosphorylation levels were reduced by 20% in PND 22 *Ccna1*^-/-^ lysates. At PND 28, this reduction reached 60% as compared to the WT type controls, while the total levels of Akt remained unchanged at all time points. To confirm that changes in Akt phosphorylation in response to depletion of Ccna1 occur specifically in the spermatocytes, we immunostained phospho-Akt (serine 473) in WT and *Ccna1*^-/-^ PND 17, PND 22 and PND 28 testes. We observed that phospho-Akt signal localized principally in spermatocytes at PND 17 WT and *Ccna1*^-/-^ testes; and PND 22 and PND 28 WT testes (Fig 4C). However, and in agreement with the immunoblot results, PND 22 and PND 28 mutant spermatocytes showed a decrease in phospho-Akt staining as compared to WT testes (Fig 4C). Concomitantly, phospho-Akt levels remain unchanged between *Ccna1*^-/-^ and WT control at PND 17 and there are few if any phospho-Akt positive spermatids in WT PND 22 and PND 28 testes. This raised the possibility that Ccna1 might modulate pro-survival signaling through Akt phosphorylation and interaction with 14-3-3 proteins.

## Summary

We have provided evidence from array analysis that meiotically arrested *Ccna1*^-/-^ spermatocytes initiate apoptosis via modulation of pro- and anti-apoptotic genes related to both the intrinsic and extrinsic signaling pathways. We did not observe detectable changes in apoptosis-related gene expression between WT and mutant testes at the stage of differentiation when Ccna1 is normally first expressed (PND 17). In contrast, pro-apoptotic genes are elevated at subsequent developmental stages in the Ccna1-deficient testes. In addition, we found the repression of anti-apoptotic genes at PND 22, which was later relieved at PND 28. We suggest as a possible explanation for this observation that at later stages, repression of anti-apoptotic genes might no longer be required to maintain the induction and progression of apoptosis in mutant spermatocytes. Immunofluorescence analysis of the initiator caspases 9 and 10 revealed that apoptosis in *Ccna1*^*-/-*^ spermatocytes was first initiated by the components of the intrinsic pathway and later may be enhanced by both intrinsic and extrinsic components. We further identified 14-3-3 proteins as an interacting partner of Ccna1 and showed that the absence of Ccna1 affects the sub-cellular distribution of 14-3-3, but not the overall levels. This distribution is important for driving cells toward survival or apoptosis, possibly by affecting the expression of pro-apoptotic genes.

Our results also showed a decrease in the phosphorylation of Akt in the absence of Ccna1, which could lead to a G2/M meiotic arrest and signaling to enter apoptosis. Our results suggest that Ccna1 may also inhibit the apoptotic functions of several proteins, including Bax and Bad, by recruiting 14-3-3 in the nucleus, and in its absence in nuclei, apoptosis signaling is activated in the arrested spermatocytes. Finally, our data indicate that the intrinsic and extrinsic apoptotic signaling pathways, as well as 14-3-3 proteins, are important features of the apoptosis triggered in the absence of a key meiotic regulator, Ccna1. Although the relevance of each apoptotic factor and pathway needs to be unraveled in future work, our results contribute to the identification of the apoptotic response that results when meiosis is defective in male germ cells.

## Supporting information

S1 Fig Differential expression of apoptotic factors in Ccna1-deficient testes relative to WT control. Scatter plot analysis showed changes in apoptotic gene expression in mutant testes compared to WT control. A; PND 17, B; PND 22, and C; PND 28 respectively. Blue dots represent down-regulated genes while red dots represent up-regulated genes. Grey dots represent genes that underwent expression changes of less than 3-fold, which was chosen as the cut off value.
(TIFF)

S1 Table Apoptosis-related genes modulated in *Ccna1*^-/-^ testes. All the genes on the array are listed, along with their Accession Number. Genes undergoing at least 3-fold increase or decrease in expression as compared to the WT control at both PND 22 and 28 are indicated in bold font. The values presented are the fold change as compared to WT control. Up-regulated genes are in red and down-regulated ones are marked in blue, respectively.
(DOCX)

S2 Table Ccna1 interacting proteins identified by FLAG-pull down followed by LC-MS-MS. Gene name and their Swiss Prot accession number were indicated. The extreme right column showed the mascot score of each individual protein.
(DOCX)
