# Supplementary figures and images for: Meiotic failure in cyclin A1-deficient mouse spermatocytes triggers apoptosis through intrinsic and extrinsic signaling pathways and 14-3-3 proteins
Source: PLoS One. 2017 Mar 16;12(3):e0173926. doi: 10.1371/journal.pone.0173926 (PMC5354389; doi:10.1371/journal.pone.0173926)

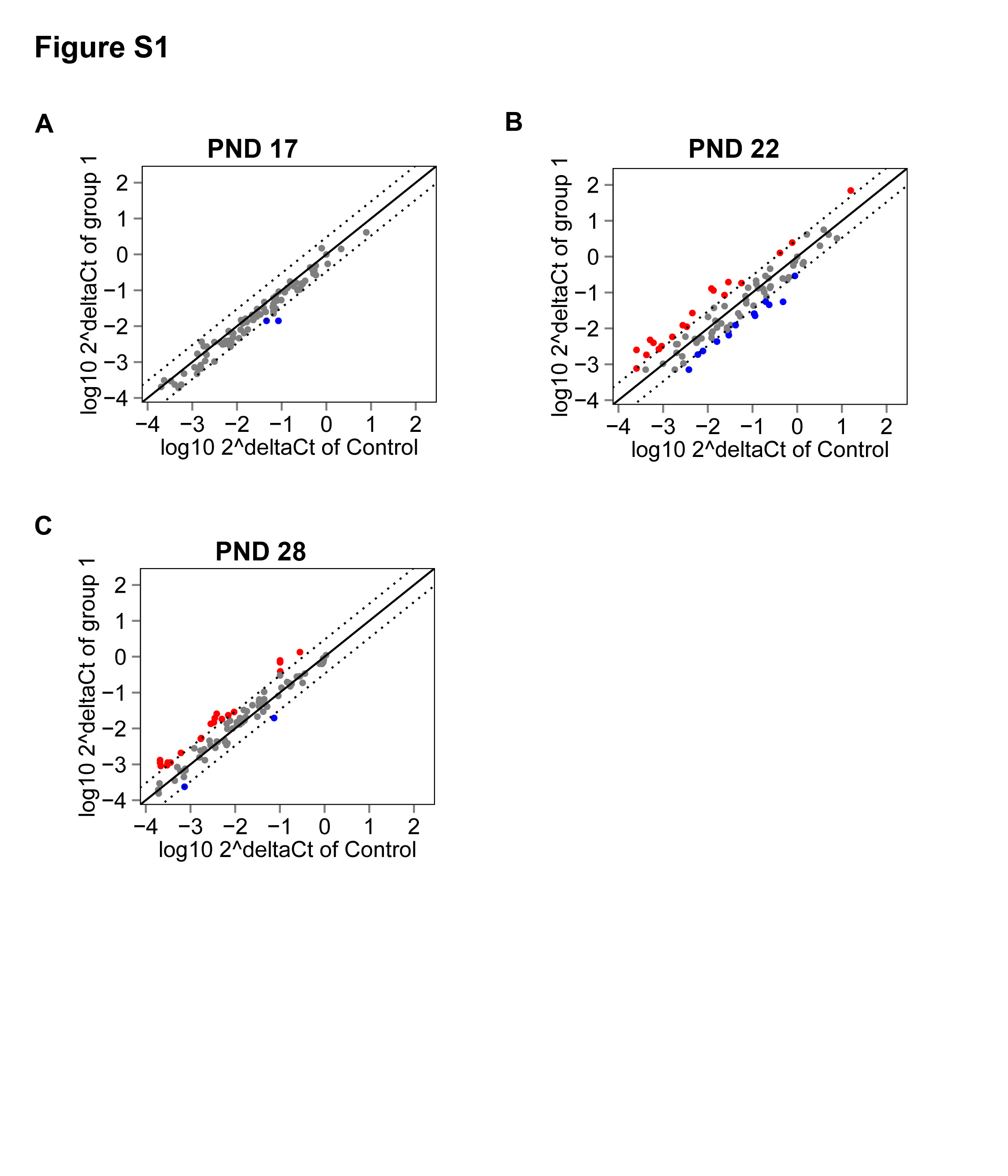

Supplement: S1 Fig — Scatter plot analysis showed changes in apoptotic gene expression in mutant testes compared to WT control. A; PND 17, B; PND 22, and C; PND 28 respectively. Blue dots represent down-regulated genes while red dots represent up-regulated genes. Grey dots represent genes that underwent expression changes of less than 3-fold, which was chosen as the cut off value. (TIFF) [file pone.0173926.s001.tiff]
