# Supplementary material for: Meiotic failure in cyclin A1-deficient mouse spermatocytes triggers apoptosis through intrinsic and extrinsic signaling pathways and 14-3-3 proteins
Source: PLoS One. 2017 Mar 16;12(3):e0173926. doi: 10.1371/journal.pone.0173926 (PMC5354389; doi:10.1371/journal.pone.0173926)
Supplement: S1 Table — All the genes on the array are listed, along with their Accession Number. Genes undergoing at least 3-fold increase or decrease in expression as compared to the WT control at both PND 22 and 28 are indicated in bold font. The values presented are the fold change as compared to WT control. Up-regulated genes are in red and down-regulated ones are marked in blue, respectively. (DOCX) [file pone.0173926.s002.docx]

| SL. No | **Gene name** | **Accession No** | **Fold Change at PND 17** | **Fold Change at PND 22** | **Fold Change at PND 28** |
| --- | --- | --- | --- | --- | --- |
| 1 | Akt1 | NM_009652 | -1.07 | 1.76 | 1.67 |
| 2 | Apaf1 | NM_009684 | -1.44 | -3.62 | 1.04 |
| 3 | Api5 | NM_007466 | -1.48 | -5.04 | -1.07 |
| 4 | Atf1 | NM_030693 | 1.2 | 2.81 | 1.53 |
| 5 | Bad | NM_007522 | -1.06 | 1.72 | 2.01 |
| 6 | Bag1 | NM_009736 | 1 | -2.21 | -1.18 |
| 7 | Bag3 | NM_013863 | 2.29 | -2.56 | 1.27 |
| 8 | Bak1 | NM_007523 | 1.54 | -4.22 | 1.04 |
| 9 | Bax | NM_007527 | 1.16 | 1.63 | 2.47 |
| 10 | Bcl10 | NM_009740 | 1.01 | -1.16 | 1.15 |
| 11 | Bcl2 | NM_009741 | 1.03 | -2.44 | 3.65 |
| 12 | Bcl2l1 | NM_009743 | -1.54 | -5.16 | 1.17 |
| **13** | **Bcl2l10** | **NM_013479** | **1.06** | **3.18** | **3.17** |
| 14 | Bcl2l2 | NM_007537 | 1.04 | 1.86 | 1.25 |
| 15 | Bid | NM_007544 | -1.26 | -1.19 | -1.4 |
| 16 | Birc2 | NM_007465 | -1.16 | -3.04 | 4.76 |
| 17 | Birc3 | NM_007464 | -1.06 | 2.97 | 2.76 |
| 18 | Birc5 | NM_009689 | -2.22 | 1.57 | 1.02 |
| 19 | Bnip2 | NM_016787 | -1.38 | 1.98 | -1.05 |
| 20 | Bnip3 | NM_009760 | -1.8 | 1.77 | 1.6 |
| 21 | Bnip3l | NM_009761 | -1.74 | -1.39 | 1.02 |
| 22 | Bok | NM_016778 | -1.81 | 2.64 | 3.06 |
| 23 | Card6 | NM_01163138 | -5.88 | 3.19 | -1.43 |
| **24** | **Card10** | **NM_130859** | **1.12** | **3.59** | **3.44** |
| 25 | Casp1 | NM_009807 | -1.69 | 1.1 | 2.32 |
| **26** | **Casp12** | **NM_009808** | **-1.57** | **6.57** | **6.31** |
| 27 | Casp14 | NM_009809 | -2.14 | 5.98 | -1.7 |
| 28 | Casp2 | NM_007610 | -2.24 | -1.53 | 1.21 |
| 29 | Casp3 | NM_009810 | -1.12 | 2.38 | 3.05 |
| **30** | **Casp4** | **NM_007609** | **-1.86** | **3.64** | **3.18** |
| 31 | Casp6 | NM_009811 | -1.44 | 1.47 | 1.21 |
| 32 | Casp7 | NM_007611 | -1.56 | -1.44 | 1.47 |
| 33 | Casp8 | NM_009812 | -1.29 | -1.33 | -1.01 |
| 34 | Casp9 | NM_015733 | -2.24 | 1.95 | 1.68 |
| 35 | Cd40 | NM_011611 | -1.71 | -2.38 | -1.26 |
| 36 | Cd40lg | NM_011616 | 1.4 | 1.6 | -3.09 |
| 37 | Cd70 | NM_011617 | -1.29 | 4.35 | 1.03 |
| 38 | Cflar | NM_009805 | -1.49 | 1.77 | 3.65 |
| 39 | Cidea | NM_007702 | 1.07 | 1.78 | 1.3 |
| 40 | Cideb | NM_009894 | 1.16 | -5.53 | -1.72 |
| **41** | **Cradd** | **NM_009950** | **-1.75** | **3.38** | **3.36** |
| 42 | Dad1 | NM_010015 | 1.29 | -3.7 | -1.23 |
| **43** | **Dapk1** | **NM_029653** | **-1.37** | **3.47** | **6.87** |
| 44 | Dffa | NM_010044 | -1.19 | -2.19 | -1.2 |
| 45 | Dffb | NM_007859 | -1.47 | -2.65 | -1.84 |
| 46 | Fadd | NM_010175 | -2.44 | 2.55 | -1.25 |
| **47** | **Fas** | **NM_007987** | **-2.24** | **10.78** | **4.2** |
| 48 | Fasl | NM_010177 | -2.6 | 3.3 | -1.6 |
| 49 | Hells | NM_008234 | -2.19 | -1.3 | -1.13 |
| **50** | **Il10** | **NM_010548** | **-2.41** | **9.95** | **6.76** |
| **51** | **Lhx4** | **NM_010712** | **-2.06** | **9.39** | **3.14** |
| 52 | Ltbr | NM_010736 | -1.51 | -4.19 | 1.84 |
| 53 | Mcl1 | NM_008562 | -1.26 | 1.15 | 1.36 |
| 54 | Naip1 | NM_008670 | -2.38 | 1.44 | -1.59 |
| 55 | Naip2 | NM_010872 | 1.32 | 2.59 | 5.36 |
| 56 | Nfkb1 | NM_008689 | -1.54 | 2.44 | 2.11 |
| 57 | Nme5 | NM_080637 | -2.9 | -1.7 | -1.22 |
| 58 | Nod1 | NM_172729 | -3.03 | 4.47 | 1.67 |
| 59 | Nol3 | NM_030152 | -1.13 | 2.03 | 1.1 |
| **60** | **Pak7** | **NM_172858** | **-2.66** | **6.79** | **3.2** |
| 61 | Pim2 | NM_138606 | -1.69 | -3.3 | 1.03 |
| 62 | Polb | NM_011130 | -2.41 | -2.62 | -1.33 |
| **63** | **Prdx2** | **NM_011563** | **-1.18** | **3.14** | **3.08** |
| 64 | Pycard | NM_023258 | -1.47 | 1.85 | 1.44 |
| 65 | Ripk1 | NM_009068 | -1.83 | -1.81 | 1.03 |
| 66 | Rnf7 | NM_011279 | -1.5 | 4.44 | -1.39 |
| **67** | **Sphk2** | **NM_203280** | **1.07** | **-8.7** | **-3.09** |
| **68** | **Tnf** | **NM_013693** | **-1.99** | **3.03** | **8.28** |
| 69 | Tnfrsf10b | NM_020275 | -1.67 | -3.19 | 1.13 |
| 70 | Tnfrsf11b | NM_008764 | -2.39 | -2.4 | -1.13 |
| 71 | Tnfrsf1a | NM_011609 | 1.01 | 1.52 | 5.56 |
| 72 | Tnfrsf10 | NM_009425 | -2.71 | -1.81 | 1.46 |
| 73 | Tnfrsf12 | NM_011614 | -1.25 | -1.28 | 4.45 |
| 74 | Traf1 | NM_009421 | -1.18 | -1.44 | 1.71 |
| 75 | Traf2 | NM_009422 | -2.15 | -1.33 | 1.22 |
| 76 | Traf3 | NM_011632 | -2.35 | -2.38 | 1.21 |
| 77 | Trp53 | NM_011640 | -1.15 | -3.52 | 2.3 |
| 78 | Trp53bp2 | NM_173378 | -1.58 | -3.38 | 1.38 |
| 79 | Trp53inp1 | NM_021897 | -1.32 | -1.67 | -1.27 |
| 80 | Trp63 | NM_011641 | 1.31 | -5.28 | 3.07 |
| 81 | Trp73 | NM_011642 | -1.8 | 8.6 | -1.01 |
| 82 | Tsc22d3 | NM_001077364 | -1.94 | -2.55 | 1.88 |
| 83 | Xiap | NM_009688 | 1.14 | 2.74 | 2.14 |
| 84 | Zc3hc1 | NM_001311086 | -1.82 | 1.17 | -1.14 |
