# Supplementary material for: Meiotic failure in cyclin A1-deficient mouse spermatocytes triggers apoptosis through intrinsic and extrinsic signaling pathways and 14-3-3 proteins
Source: PLoS One. 2017 Mar 16;12(3):e0173926. doi: 10.1371/journal.pone.0173926 (PMC5354389; doi:10.1371/journal.pone.0173926)
Supplement: S2 Table — Gene name and their Swiss Prot accession number were indicated. The extreme right column showed the mascot score of each individual protein. (DOCX) [file pone.0173926.s003.docx]

| SL No | Gene name | NCBI or Swissprot Accession | Masscot Score |
| --- | --- | --- | --- |
| 1 | Peptidyl-prolylcis-trans isomerase A | PPIA_Mouse | 306 |
| 2 | Ig kappa chain C region | IGKC_Mouse | 278 |
| 3 | Ig kappa chain V-II region 26-10 | KV2A7_Mouse | 167 |
| 4 | 14-3-3 protein zeta/delta | 1433Z_Mouse | 149 |
| 5 | Phosphatidylethanolamine-binding protein | PEBP1_Mouse | 132 |
| 6 | L-lactate dehydrogenase | LDHC_Mouse | 121 |
| 7 | Tubulin beta-5 chain | TBB5_Mouse | 90 |
| 8 | High mobility group protein B2 | HMGB2_Mouse | 81 |
| 9 | Tubulin beta-3 chain | TBB3_Mouse | 81 |
| 10 | Tubulin alpha-1A chain | TBA1A_Mouse | 74 |
| 11 | Small ubiquitin-related modifier 2 | SUMO2_Mouse | 72 |
| 12 | Protein disulfide-isomerase A6 | PDIA6_Mouse | 68 |
| 13 | 14-3-3 protein theta | 1433T_Mouse | 62 |
| 14 | Calreticulin | CALR_Mouse | 62 |
| 15 | Cyclin-A1 | CCNA1_Mouse | 57 |
| 16 | D-dopachrome decarboxylase | DOPD_Mouse | 56 |
| 17 | Stathmin | STMN1_Mouse | 55 |
| 18 | Glutathione S-transferase Mu 1 | GSTM1_Mouse | 52 |
| 19 | Endoplasmin | ENPL_Mouse | 43 |
| 20 | Superoxide dismutase [Cu-Zn] | SODC_Mouse | 37 |
| 21 | Hydroxyacylglutathione hydrolase, mitochondrial | GLO2_Mouse | 36 |
| 22 | Nuclear autoantigenic sperm protein | NASP_Mouse | 35 |
| 23 | Heat shock 70 kDa protein 4L | HS74L_Mouse | 32 |
| 24 | Phosphoglyceratemutase 2 | PGAM2_Mouse | 30 |
| 25 | 78 kDa glucose-regulated protein | GRP78_Mouse | 367 |
| 26 | Heat shock protein HSP 90-beta | HS90B_Mouse | 138 |
| 27 | Pyruvate dehydrogenase E1 component subunit beta | ODPB_Mouse | 98 |
| 28 | Elongation factor 1-alpha | EF1A2_Mouse | 81 |
| 29 | Hsc70-interacting protein | F10A1_ Mouse | 66 |
| 30 | Programmed cell death protein 5 | PDCD_Mouse | 54 |
